# Are disease-specific patient-reported outcomes measures (PROMs) used in cardiogenetics? A systematic review

**DOI:** 10.1038/s41431-023-01510-w

**Published:** 2023-12-14

**Authors:** Saar van Pottelberghe, Nina Kupper, Esther Scheirlynck, Ahmad S. Amin, Arthur A. M. Wilde, Nynke Hofman, Edward Callus, Ruth Biller, Julie Nekkebroeck, Sonia Van Dooren, Frederik J. Hes, Saskia N. van der Crabben

**Affiliations:** 1 Clinical Sciences, Research Group Reproduction and Genetics, Centre for Medical Genetics, Universitair Ziekenhuis Brussel (UZ Brussel), Vrije Universiteit Brussel (VUB), Brussels, Belgium; 2 Member of the European Reference Network for Rare, Low Prevalence, and/or Complex Diseases of the Heart: ERN GUARD-Heart, Amsterdam, The Netherlands; 3 Center of Research on Psychological Disorders and Somatic Diseases; Department of Medical & Clinical Psychology, Tilburg University, Tilburg, the Netherlands; 4 Cardiology Department, Universitair Ziekenhuis Brussel—Vrije Universiteit Brussel, Brussels, Belgium; 5 Department of Cardiology, Amsterdam UMC Location University of Amsterdam, Amsterdam, the Netherlands; 6 Amsterdam Cardiovascular Sciences, Heart Failure and arrhythmias, Amsterdam, the Netherlands; 7 Clinical Psychology Service, IRCCS Policlinico San Donato Research and University Hospital, San Donato Milanese, Milan, Italy; 8 Department of Biomedical Sciences for Health, University of Milan, Milan, Italy; 9 European Patient Advocacy Group of the European Reference Network for Rare, Low Prevalence, and/or Complex Diseases of the Heart: ERN GUARD-Heart, Amsterdam, The Netherlands; 10 ARVC-Selbsthilfe e.V., ARVC Patient Association, Munich, Germany; 11 Clinical Sciences, Research Group Reproduction and Genetics, Brussel Interuniversity Genomics High Throughput Core (BRIGHTcore), Universitair Ziekenhuis Brussel (UZ Brussel), Vrije Universiteit Brussel (VUB), Brussels, Belgium; 12 Department of Clinical Genetics, Amsterdam UMC, University of Amsterdam, Amsterdam, the Netherlands

**Keywords:** Quality of life, Health services

## Abstract

Patient-reported outcome measures (PROMs) are used to facilitate patient-centered care (PCC). While studies in patients with cardiac conditions have revealed poorer health-related quality of life (HRQoL) and elevated emotional stress, studies in inherited cardiac conditions (ICC) seem rare. A systematic review evaluated which (specific domains of) PROMs are used in patients with ICC. From three databases (PubMed, PsychINFO, and Web of Science) quantitative studies investigating PROMs in patients with ICC were included. A Cochrane-based assessment tool was used to evaluate quality and potential risk of bias per subdomain. Data from 17 eligible articles were extracted. Among the included studies, risk of bias was predominantly high (35%) or unclear (30%). Most (*n* = 14) studies used a generic health status measure (SF-36, SF-12); 3 studies used a disease-specific PROM (KCCQ- cardiomyopathy and MLFHQ-heart failure). In addition to HRQoL measures, several studies used affective psychological measures (i.e., HADS, CAQ-18, IES-R, and IPQ). The mental health component of the PROMs showed lower scores overall in patients with ICC compared to population norms. Nine studies using HADS and GAD-7/PHQ-9 showed a prevalence of clinically significant anxiety (17–47%) and depression levels (8.3–28%) that were higher than the population norm (8.3% and 6.3%, respectively). HRQoL in patients with ICC is primarily assessed with generic PROMs. Results further confirmed high psychological morbidity in this population. Generic PROMS measures evaluate overall health status, but lack sensitivity to ICC-specific factors like heredity-related concerns. We propose developing a PROM specific for ICC to optimize PCC.

## Introduction

Health-related quality of life (HRQoL) is a crucial outcome for patients with a chronic heart condition. I.e., it enables the assessment of the impact of the heart condition on physical and mental well-being and has impact on complex medical outcomes [1]. HRQoL can be affected by disease-related consequences, such as the necessity for immediate lifestyle changes, the effects of pharmacotherapy, or more invasive procedures like radiofrequency ablation and the placement of an implantable cardioverter-defibrillator [2, 3]. Furthermore, experiencing symptoms such as syncope, palpitations, dyspnea, chest pain, and life-threatening arrhythmias can limit a patient’s well-being physically as well as mentally [4–6]. Elevated psychological distress (i.e., anxiety, depression) has already been shown in patients with ischemic heart disease, heart failure, primary arrhythmias, and cardiomyopathies [7–11].

Patient-reported outcome measures (PROMs) are standardized questionnaires to gauge patients’ subjective reports of how they feel and function. In today’s health system, measuring outcomes that are most important to patients is vital. Patient-reported outcomes (e.g., self-report, no interpretation needed) and clinician-reported (e.g., interpreted by clinician) functional outcomes measure different components of perceived patient wellbeing. PROMs can be used for assessing HRQoL, e.g., the personal impact of illness, treatment, and clinical interventions [12], and can be a metric for delivering high-quality cardiogenetic care [11]. PROMs play an essential role in understanding patients’ experiences of health conditions and debilitating consequences by recognizing psychosocial factors and implementing and delivering patient-centered care [13, 14].

The perspective of patients is crucial in inherited cardiac conditions (ICC), where patients cope with the complex facts of living with a chronic disease with aggravation and possible various cardiac treatments (e.g., need for an ICD or beta-blockers versus surveillance and surgery), together with heredity related concerns (e.g., fear of inheriting a disease; worry family members might be affected). The physical and psychological challenges faced by patients and their “at-risk” relatives with ICC can affect their HRQoL, psychological well-being, treatment adherence, and medical outcomes [4]. Primary up to tertiary care providers should be mindful of these challenges and unique factors to lower psychological morbidity in patients with ICC.

PROMs used in oncogenetics have shown that, apart from the disease-associated worries, patients with an inheritable disease can also experience problems with adjustment related explicitly to the process of genetic testing, where factors like heredity, reproductive choices, and concerns for family members come into play [15–17]. Furthermore, the heredity pattern of ICC is complex: mainly autosomal dominance with incomplete penetrance and variable expression of these diseases. This means that a substantial proportion of “at-risk” patients will never manifest the disease [18, 19]. Because of this uncertainty, disclosing positive test results can raise feelings of ambiguity and anxiety [20]. Specific hereditary worries (i.e., extended family risks, the immediate risk for carriers irrespective of age, incomplete penetrance and variable expression of disease, and the threat to minor children) can develop throughout the process of genetic testing [21–23].

Until now, research assessing the impact of genetic testing focused mainly on affective outcomes, e.g., general distress, anxiety, depression, and worry [24, 25]. In addition questionnaires administered in a research setting or the genetic clinic cover *satisfaction of counseling* or *care* (patient-experience measures (PREMs)) but do not address heredity and specific ICC-associated complaints [26–28]. In contrast to a generic measure, which can be useful at organizational levels (e.g., compare cost-effectiveness of interventions in different patient groups, or compare the overall evaluation of care), disease-specific PROMs are designed to assess treatment outcomes at patient level. Additional research on these specific topics is therefore essential to improve care of ICC patients.

## Methods

The protocol for this review was registered with PROSPERO (#CRD42021271384) in September 2021. In March 2023, a revision note was added to the registered protocol explaining why we deleted one of the research questions. We adhered to the PRISMA guidelines for reporting systematic reviews.

### Eligibility criteria

Quantitative studies investigating PROMs in patients with ICC were included. PROMs are defined as the uninterpreted and self-reported experience of a patient’s health, functional status, and HRQoL associated with health care or treatment [28]. Only articles written in English were included.

Exclusion criteria comprised reports regarding cardiovascular conditions without a specific genetic basis, systematic reviews, qualitative studies, or case studies. We excluded studies with a pediatric ICC population (<15 y), because these studies report on proxy-PROMs completed by the caregivers and are therefore not actually “patient-reported”, but merely mirror the caregiver’s feelings and anxieties. Further, studies reporting PROMs for cardiac conditions other than ICC (i.e., atrial fibrillation, congenital cardiac disease) and articles whose primary aim was to investigate the influence of an implanted cardiac defibrillator (ICD) on aspects of patient-reported outcomes were omitted.

### Search strategy

Nine authors (ASA, AAW, EC, ES, FH, NK, RB, SvdC, and SVP) and a medical librarian (KA) were involved in the composition of the search terms. The search strategy comprised three terms: population, PROMs, and genetic screening. The search comprised three blocks of terms relating to health status, cardiac arrhythmias, and genetic testing. Search terms were developed for each key domain of the research question and are set as follow:

Population: patients with inherited cardiovascular disease. We used the following keywords to identify eligible studies: cBrugada syndrome”, “sudden unexplained death syndrome”, “right bundle branch block”, “Long QT syndrome”, “Catecholaminergic Polymorphic Ventricular Tachycardia”, “cardiomyopathies”, “hypertrophiccardiomyopathy”, “dilated cardiomyopathies” and “arrhythmogenic cardiomyopathy”.

Outcome: the medical outcomes of interest for the rapid review are the impact of PROMs on “health status”, “quality of life” and “health-related quality of life”. The “Genetic screening” subgroup is used as a third block and comprised of the keywords “genetic” and served as a bottleneck.

We used the Boolean operator “OR” to combine the terms in each domain and the Boolean operator “AND” to connect the concepts under research.

### Data extraction

In the primary screening stage, three authors (SVP, ES, and ASA) independently screened titles and abstracts of the retrieved studies in Covidence using the predetermined eligibility criteria. Covidence is a screening and data extraction tool for conducting systematic reviews. This online platform allows more efficient screening. For the full-text review stage, we recorded reasons for exclusion. Then, for each study, two authors (SVP and SvdC) independently undertook data extraction using the default data extraction template to reduce errors. Data extraction included study aims, location, setting, study design, sample characteristics, PROM used/implemented, outcomes, and results.

### Quality assessment

Covidence uses customizable quality assessment (QA) forms. We used a previously validated critical appraisal risk of bias tool [26], which is presented as online Supplemental File S1). Two raters (SVP and SvdC). We documented reasons for judgments and resolved discrepancies by discussing or consulting a third rater (NK). Following Cochrane’s recommendations [29], study quality was assessed according to the following domains of potential bias: (a) selection bias, (b) attrition bias for prospective studies, (c) information bias, (d) reporting bias, and (e) lack of precision.

Per domain each criterion is evaluated for a judgment, “yes”, “no”, or “unclear”. Per type of bias, a low risk of bias is assigned if all criteria are judged with “yes”. In this case, it is unlikely that plausible bias will seriously alter the study results. A high risk of bias is assigned when the criteria are judged “no”, meaning alleged bias seriously weakens confidence in the study results. If one or more criteria are judged with “unclear” but one with “yes”, “unclear” risk of bias is assigned. In this case, some doubt about the study results is raised. The overall potential risk of bias summary score was defined as “low”, “high”, or “unclear” risk of bias for one or more key domains to assess summary outcome (across bias domains) for each included study.

### Data analysis and reporting

In a narrative synthesis, we report and structure the findings of the included studies around the type of PROM used, population characteristics, psychosocial dimensions, and physical and medical variables used in cardiogenetics. We report on which measures are used to assess overall self-rated health status and report on the impact of ICC on various outcomes in a narrative analysis.

Different assessment tools can measure this: PROMs are used to gain insight into the patient’s perspective on how aspects of their health and the impact of the disease and its treatment influence their lives. PROMS may include questionnaires that measure constructs like health status/HRQoL (example questions: “I can walk the stairs”, “To what extent did your health hinder work activities”), QoL (e.g., “How satisfied are you with your health”, “Do you feel you have enough energy for everyday life”), and symptoms (e.g., dizziness, fatigue, chest pain) and the scores can be used to improve the quality of patient care [27]. The scope of PROMS can be generic or disease-specific, where generic PROMS are helpful when comparing patients across health conditions, or with the general population, these questionnaires lack sensitivity to disease-specific outcomes. In contrast, disease-specific PROMS track specific aspects of a disease and, therefore, have higher face validity (e.g., whether a test appears to measure what it is supposed to measure) and show better responsiveness to patient disease [30].

PREMS gathers information on patients’ views of their experience with the care received and differ from PROMs, as these questionnaires do not look at care outcomes but focus on the care process[12]. Finally some studies report on the emotional state of the patients, including anxiety, depression, coping, well-being, and adjustment. Assessed with their own set of instruments like the HADS, PHQ-9 (example question: “Over the past two weeks, I had little interest or pleasure in doing things”). These surveys typically assess the presence of (pre)clinical depression and anxiety, and one should discriminate them from measures assessing health status/HRQoL/QoL [31].

## Results

### Study selection

**Fig. 1:**
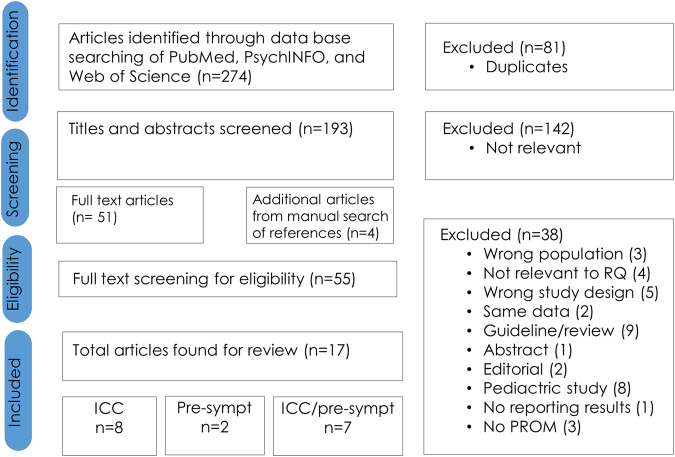
Flowchart study-selection process. This figure displays the selection process of the articles selected for the review. Abbreviations used in Fig. 1, inherited cardiac conditions (ICC); presymptomatic carrier (pre-sympt); research question (RQ).

Figure 1 illustrates the flowchart of the inclusion and selection process. In summary, the electronic databases PubMed, PsychINFO, and Web of Science generated 274 citations. We excluded 261 articles, and we added 4 through snowballing, leaving 17 articles for inclusion.

We listed the study characteristics in Table 1. The samples in the reviewed articles were predominantly male, white, highly educated population, with a mean age of 45 years, and two third of participants were married or in a relationship. The 17 included studies covered PRO reports from 2648 participants, with sample sizes ranging from 24 to 486. Population characteristics varied in terms of sex distribution, age, clinical diagnosis, genetic testing (pre-symptomatic, symptomatic- and diagnostic screening), carrier status, and “at-risk” family members. Eight studies had a population with a clinical diagnosis of ICC [hypertrophic cardiomyopathy (HCM), dilated cardiomyopathy, non-compaction cardiomyopathy]; seven studied a population with a clinical diagnosis of ICC and their “at-risk” family members [long QT syndrome, HCM, catecholaminergic polymorphic ventricular tachycardia], and two studied the impact of family-based screening in pre-symptomatic patients with ICC [arrhythmogenic cardiomyopathy].

**Table 1:** Study characteristics.

Study ID	Sample size (*n*)	Year	Study design	Country	Disease	Population	RQ 1	RQ 2
Brouwers 2015	130	2015	CROSS-SECTIONAL	THE NETHERLANDS	NCCM	Manifest ICC	- health status	- adverse psychological factors
Hamang 2010	127	2010	CROSS-SECTIONAL	NORWAY	LQTS, HCM; AR HCM, AR LQTS	Manifest ICC + presymptomatic	- health status	- sociodemographic determinants & health status domains
Ingles 2013	409	2013	CROSS-SECTIONAL	AUSTRALIA	ICC and AR ICC	Manifest ICC + presymptomatic	- health status	N/A
Hamang 2011	126	2011	CROSS-SECTIONAL	NORWAY	LQTS, HCM; AR HCM, AR LQTS	Manifest ICC + presymptomatic	- HFA	N/A
Hamang 2012	173	2012	PROSPECTIVE	NORWAY	LQTS, HCM; AR HCM, AR LQTS	Manifest ICC + presymptomatic	- HFA	N/A
Cox 1997	137	1997	CROSS-SECTIONAL	UNITED KINGDOM	HCM	Manifest ICC	- HRQOL	- PW
Steptoe 2000	60	2000	CROSS-SECTIONAL	UNITED KINGDOM	DCM	Manifest ICC	- HRQOL	- PD
Christiaans 2009	228	2009	CROSS-SECTIONAL	THE NETHERLANDS	HCM	Manifest ICC	- HRQOL	- sociodemographic, risk and illness perception
Pedrosa 2010	84	2010	CROSS-SECTIONAL	BRAZIL	HCM	Manifest ICC	- HRQOL	- sleep
Huff 2012	24	2012	CROSS-SECTIONAL	USA	HCM	Manifest ICC	- HRQOL	- correlation between KCCQ and NYHA class
Capota 2020	91	2020	CROSS-SECTIONAL	ROMANIA	HCM	Manifest ICC	- HRQOL	- identify predictors
Ingles 2012	54	2012	PROSPECTIVE	AUSTRALIA	ICC ; AR ICC	Manifest ICC + presymptomatic	- HRQOL	N/A
Richardson 2018	54	2018	CROSS-SECTIONAL	AUSTRALIA	CPVT; AR CPVT	Manifest ICC + presymptomatic	- HRQOL	- PW
McGorrian 2013	334	2013	CROSS-SECTIONAL	IRELAND	AR ICC	Presymptomatic	- HRQOL	- PW
Brothers 2021	73	2021	PROSPECTIVE	CANADA	AR ARVC	Presymptomatic	- HRQOL	- PD
Hickey 2014	58	2014	CROSS-SECTIONAL	USA	ICC	Manifest ICC	- PD/PW	N/A
Ingles 2015	486	2015	CROSS-SECTIONAL	AUSTRALIA	HCM; AR HCM	Manifest ICC + presymptomatic	- PD/PW	- sociodemographic determinants
Total	***N*** = **2648 [24–486]**	**[1997–2021]**						

This table shows the study characteristics (study design, country, and sample size) and primary outcome measures used of all included studies. The 17 studies included PRO reports from 2648 participants, with sample sizes ranging from 24 to 486. Population characteristics varied in terms of sex distribution, age, clinical diagnosis, genetic testing (pre-symptomatic, symptomatic and diagnostic screening), carrier status, and “at-risk” family members.

*NCCM* noncompaction cardiomyopathy, *LQTS* long QT syndrome, *HCM* hypertrophic cardiomyopathy, *AR* at risk, DCM dilated cardiomyopathy, *CPVT* catecholaminergic polymorphic ventricular tachycardia, *ARVC* arrhythmogenic right ventricular cardiomyopathy, *PD* psychological distress, *PW* psychological wellbeing, *HFA* heart-focused anxiety.

### Quality assessment and risk of bias

**Fig. 2:**
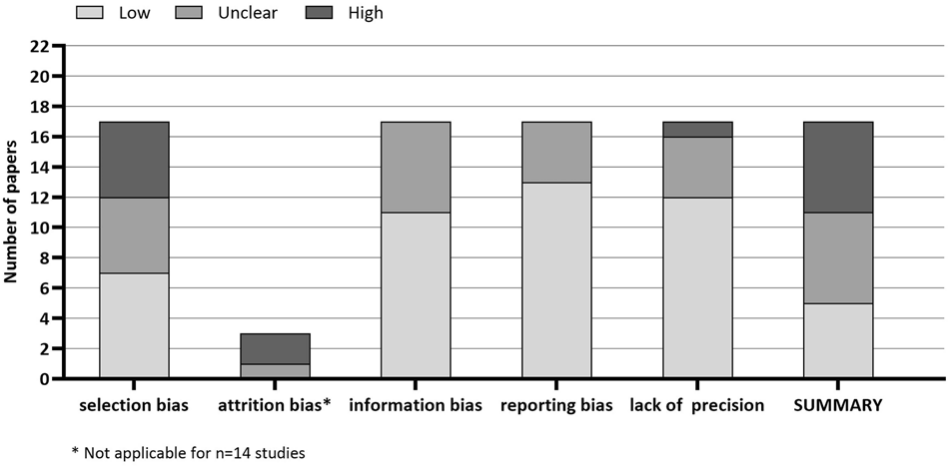
Risk of Bias-summary. Note: This figure displays the sum of all risk of bias for each included study. Light gray = potential bias is low; middle gray = potential bias in this domain is unclear (plausible bias that raises some doubt about the results of this study); dark gray = potential bias is high (plausible bias that seriously weakens confidence in the results of this study). Attrition bias is only applicable in prospective studies (*n* = 3).

Figure 2 displays a summary of all risk of bias for each included study. We found that the risk of Bias across all studies was mainly high (35%) or unclear (30%). Especially selection bias (59%) showed high/unclear risk. About 35% of the articles did not mention a response rate, and another 27% had a response rate lower than 60%). Three of the 17 studies lacked inclusion criteria, and eight did not describe exclusion criteria. Figure S1 details the summary of rated biases for all the studies included.

### Data synthesis

#### Patient-reported outcome measures

**Fig. 3:**
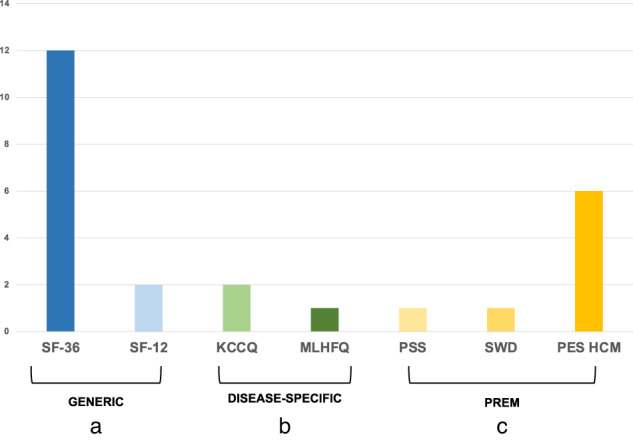
PROMS/PREMS in cardiogenetics. This figure shows the percentage generic PROMs used (82%) and the disease-specific PROMs used (18%). **a** Generic PROMs: the MOS Short Form 36 (SF-36) or its briefer form, the SF-12. **b** Disease-specific: the Kansas City Cardiomyopathy Questionnaire (KCCQ); the Minnesota Living with Heart Failure Questionnaire (MLFHQ). In addition to PROMs, some studies used an unvalidated patient-reported measure (47%) together with generic PROM. PREM the patient satisfaction scale (PSS), the satisfaction with decision scale (SWD), the patient experience scale for HCM (PES HCM).

Table S2 shows all the PROMs, PREMs, and affective measures used in the included studies of the review. The selected studies used different instruments (combined or alone) to gauge patients’ overall self-rated health status (Fig. 3). Of the 17 studies, 14 used a generic PROM (82%), like the MOS Short Form SF-36 or its briefer form, the SF-12 [32, 33]. Three studies (18%) used a disease-specific PROM focusing on HRQoL in patients with cardiovascular disease, such as a questionnaire specific for cardiomyopathy, the Kansas City Cardiomyopathy Questionnaire (KCCQ) [34, 35] and a questionnaire specific for heart failure, the Minnesota Living with Heart Failure Questionnaire (MLFHQ) [36]. None of the PRO instruments were validated for use in patients with ICC specifically.

Several studies (*n* = 14) used the physical component score of the SF-36/SF-12 [2, 3, 9, 18, 19, 32, 33, 37–43], and the physical limitation score of the KCCQ [34, 35].

#### Patient-reported experience measures

In addition to the PROMs, eight studies (47%) used a PREM, such as the patient satisfaction scale [38], the satisfaction with decision scale [9], and the patient experience scale for HCM [9, 18, 33, 38, 39, 43] a not yet validated PREM.

#### Psychological survey

Several studies (*n* = 11) used affective psychological measures to identify subgroups at risk for developing high levels of psychological distress at different time points in the process of a genetic risk assessment.

**Fig. 4:**
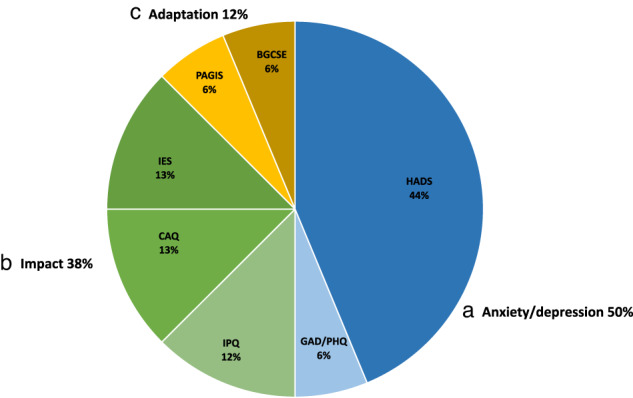
Psychological surveys. This figure shows the additional psychological measures that studies used to measure psychological well-being. **a** Anxiety and depression surveys [blue color]:: Hospital Anxiety and Depression Score (HADS); Generalized Anxiety Disorder Scale (GAD-7); Patient Health Questionnaire (PHQ-9); **b** Impact scales [green color]: Impact of Event Scale (IES-15; IES-R); Cardiac Anxiety Questionnaire-18 (CAQ-18); The Illness Perception Questionnaire-revised (IPQ-R); **c** Adaptation scales [yellow color]:: The Bergen Genetic Counselling Self-Efficacy scale (BGCSE); Psychological adaptation to genetic information scale (PAGIS).

Figure 4 shows that 50% of the studies used a screener for anxiety/depression, like the HADS [9, 18, 33, 38, 39, 42, 43] and the PHQ-9 combined with the GAD-7 [32]. Thirty-eight percent of the studies measured the impact of the disease with the CAQ-18 [3, 19]; IES-15/IES-R [37, 42], and the IPQ [18, 39].

In addition, two studies measured psychological adaptation when undergoing cardiac genetic screening with the PAGIS [42] and Bergen genetic counseling self-efficacy scale questionnaire [19].

A narrative analysis of various outcomes is presented below and is discussed in more detail in the subsequent section.

#### Summary of the findings

The main findings of the studies on HRQoL, and psychological well-being are listed in Tables 2 and 3. Overall, study scores of patients with a clinical (but no genetic) diagnosis of ICC showed significant limitations on all health domains of the SF-36 compared to the general population [2, 18, 19, 32, 38, 42, 43]. Symptomatic patients with a clinical and genetic diagnosis of ICC reported lower HRQoL than genetic carriers of familial ICC without clinical symptoms [32]. Two cross-sectional studies showed decreases in all disease-specific KCCQ domains for patients with clinical (but no genetic) HCM [34, 35], and a significant reduction in the quality of life domain of the MLHFQ (mean score 20, range [11–40]) was found [36]. Notably. A high-quality study used the generic SF-12 to measure HRQoL and found no association between HRQoL, the screening indication and ICC type (i.e., primary arrhythmia syndrome vs. cardiomyopathies vs. sudden arrhythmic death syndrome) [33].

**Table 2:** Main findings of PROMs.

Study ID	Sample	Measure	Measure Outcome	Overall Findings	Findings physical health component (PCS)
Cox 1997	HCM; *N* = 137	SF-36	GENERIC	- Symptom pattern strongly associated with HRQoL and wellbeing; - Patients with dyspnoe reported poorer HRQoL. - HRQoL also associated with patterns of chest pain.	- Patients who experienced both atypical and exertional pain had the lowest scores on physical functioning, role limitations due to physical factors, general health perceptions, vitality and bodily pain. - Syncope →significantly lower PCS and bodily pain scales
Steptoe 2000	DCM; *N* = 60	SF-36	GENERIC	- Pronounced restrictions in QoL and wellbeing; - Major limitations on all SF-36 dimensions except bodily pain; - In comparision with HCM pop: DCM sign greater restrictions in social functioning; HCM experience sign higher levels of pain - Several aspects of impaired HRQoL are not simple functions of disease severity, but determined by factors related to psychological adjustment as well.	- Older patients experience more vitality; female sign poorer HRQoL than male in terms of physical funtioning; perc gen health and bodily pain; pat with lower shortening fractions had poorer HRQoL PCS - DCM fam reported better phys funct, fewer role lim.
Christiaans 2009	HCM; *N* = 228	SF-36	GENERIC	- Carriers HCM report ↓HRQoL	- Carriers HCM: ↓ general health perception; ↓vitality, ↓bodily pain - Subgroup carriers with manifest HCM sign worse physical prediction
Hamang 2010	LQTS, HCM + AR LQTS, AR HCM; *N* = 127	SF-36	GENERIC	- Manifest HCM: scored lower on all health domains compared to patients with LQTS-syndrome or with patients at risk. - ↑ Manifestations/symptoms to affect health	- Physical health differed according to disease status - Manifest HCM, LQTS report poorer physical health - AR patients reported better phys health
Pedrosa 2010	*N* = 126; HCM (*n* = 84); asymptomatic controls (*n* = 42)	MLHFQ	DISEASE-SPECIFIC	- Poor HRQoL was independently associated with poor sleep quality, NYHA classification and diuretic therapy. - Congestive heart failure symptoms ass worse HRQoL	- No results on physical health
Hamang 2011	LQTS, HCM + AR LQTS, AR HCM; *N* = 126	SF-36	GENERIC	N/A	- ↑ Manifestations/symptoms to affect health
Ingles 2012	ICC (33) + AR ICC(21); *N* = 54	SF-36	GENERIC	- No change HRQoL, - No change in health status outcome due to genetic testing	- HCM, DCM, CPVT → ↓ PCS - AR HCM and carriers HCM; AR LQTS; AR DCM→ ↑ PCS
Ingles 2015	HCM; *N* = 486	SF-36	GENERIC	- Predictive genetic test for adult onset disease does not lead to long term impairment of HRQoL	- ↑ comorbidities; ↓ income; ↓ specialized clinic
Brouwers 2015	NCCM(45), FH(43), acq DCM (42); *N* = 130	SF-12	GENERIC	- Stronger adverse eff on health status in patients with manifest HCM LQTS versus AR patients without symptoms	- ↑ comorbidity, ↑ ICD implant ↓ age, ↓ education
Richardson 2018	CPVT; *N* = 54	SF-36	GENERIC	- Vitality (energy and fatigue)domain: worse than gen pop	- No results on physical health
Hamang 2012	LQTS, HCM + AR LQTS, AR HCM; *N* = 173	SF-36	GENERIC	N/A	- Physical health differed according to disease status. - Patients with clinical LQTS or clinical HCM reported poorer physical health - Higher avoidance (measured with HFA) is strongly related to poorer physical health
Huff 2013	HCM; *N* = 24	KCCQ	DISEASE-SPECIFIC	- KCCQ results demonstrated moderate reductions in all domains, with greatest reduction in HRQoL-domain	- KCCQ (physical functioning and overall scores) has moderate correlation with NYHA class and cardiopulmonary exercise test
McGorrian 2013	AR ICC; *N* = 316	SF-12	GENERIC	- No association with a difference in HRQoL found when comparing different patients groups (e.g., SADS vs channelopathy vs cardiomyopathy)	- PCS-12 decreased with age (p = 0.01), indicating worsening physical health
Ingles 2013	ICC; *N* = 409	SF-36	GENERIC	- HCM, DCM, CPVT→ ↓ PCS - AR HCM and carriers HCM; AR LQTS; AR DCM→ ↑ PCS - Presence and severity of symptoms; activity restrictions; adverse medication side effects have negative effect on health status, anxiety, and depression	- Women; comorbidity; NYHA functional class]≈ impaired physical score - ↓PCS score: physical disability due to lifestyle modification recommendations - HCM, DCM, CPVT: ↓PCS vs AR HCM; AR LQTS - AR DCM and carriers HCM: ↑PCS
Hickey 2014	LQTS, BrS, HCM, DCM; *N* = 58	SF-36	GENERIC	- Similar to other studies. - No LT negative consequences of undergoing gen test - ICD-shock→ ↓ HRQoL	- Individuals with positive genetic cardiac test scored significantly lower on the bodily pain dimension
Capota 2020	HCM; *N* = 91	KCCQ	DISEASE-SPECIFIC	- Sign worse HRQoL in the HCM population as compared to matched patients from the general public. - Pulmonary hypertension significantly impact global HRQoL.	- Important gender differences in global KCCQ, poorer and higher symptom burden among women - Similar results as Hamang 2010
Brothers 2021	AR ARVC; *N* = 73	SF-36	GENERIC	- No significant differences in the scores between baseline, 6 M, 1Y	- No results on physical health

This table lists all findings/results found in the included studies from PROMs.

*NCCM* Noncompaction Cardiomyopathy, *LQTS* Long QT syndrome, *HCM* hypertrophic cardiomyopathy, *AR* at risk, *DCM* dilated cardiomyopathy, *CPVT* catecholaminergic polymorphic ventricular tachycardia, *ARVC* arrhythmogenic right ventricular cardiomyopathy, *PCS* physical health component, *PW* psychological wellbeing, *PD* psychological distress, *HFA* heart-focused anxiety.

**Table 3:** Main findings of the studies on psychological wellbeing, general anxiety/depression.

Study ID	Sample	Measure	Measure outcome	Overall findings	Predictors identified
Cox1997	HCM; *N* = 137	HADS	PW	- HADSa 8.23; 21.2% classified as possible-28.5% as probable. - HADSd 5.29; 13.1% possibles-9.5% probable; a large proportion showed considerable worry about condition; - No chest pain 4.11; atypical chest pain 5.0; patients with exertional pain 6.05; exertional and atypical pain 7.5. raised levels of anxiety 49.7%	- Chest pain associated with depression
Steptoe 2000	DCM; *N* = 60	HADS	PD	- DCM better physical functioning perceived fewer role limitations - Conclusion: this finding DOES NOT support the notion that knowledge of familial element has adverse psychological effects	- The level of psychological adjustment to CMP associated with poor physical functioning, mental health and emotional distress
Christiaans 2009	HCM; *N* = 228	HADS; Perceived risk score;IPQ	PD	- Carriers HCM: ↓general health perception; ↓vitality, ↓bodily pain - Subgroup: carriers with manifest HCM report ↓HRQoL; high PD	- Experiencing Symptoms - Higher perceived risk of symptoms contributes to poorer mental health outcomes
Hamang 2010	LQTS, HCM + AR LQTS, AR HCM; *N* = 127	SF-36 MCS	Not defined	- LQTS: ↑worries/uncertainties around symptoms, diagnosis, and disease management→PD ↑, ↓mental health:	- Women - Being referred by a physician - Having children - Lower education
Pedrosa 2010	*N* = 124; HCM (*n* = 84); asymptomatic controls (n = 42)	N/A	N/A	- 1/3 HCM→anxiety - 1/5 HCM→mood disorder	N/A
Hamang 2011	LQTS, HCM + AR LQTS, AR HCM; *N* = 126	HADS, CAQ-18	Anxiety, depression; Heart-focused anxiety	- Manifest HCM/LQTS : ↑ HFA scores (compared to at risk) - ↑levels of anxiety as compared to general public (25% clin anxiety level; 13,5% clinical depression level) - ↑general anxiety levels among the patient group - No significant differences were found in patient with LQTS or patients with familial HCM in comparing general anxiety and depression scores - Fear about heart sensations is associated to general anxiety and depression	- HFA is predictor for PD - Clinical diagnosis - Increasing age - Perceived risk of symptoms/disease
Hamang 2012	LQTS, HCM + AR LQTS, AR HCM; *N* = 173	CAQ-18;BGCSE	Heart-focused anxiety	- poorer perceived health ↑levels of self-efficacy expectations: gen testing causes disease related anxiety - Avoidance and fear significantly related to general anxiety and depression in patients with HCM/LQTS - Procedural satisfaction with genetic counseling was associated with lower levels of fear and avoidance	- Women →↑levels of fear; +gen test→ ↑ avoidance - Poor perceived health, and a family history of SCD ≈ anxiety; HFA
Ingles 2012	ICC (33) + AR ICC(21); *N* = 54	SF-36 MCS/	Not defined	- no change in psychosocial outcome due to genetic testing	N/A
Huff 2013	HCM; *N* = 24	N/A	N/A	- No results on anxiety and depression	N/A
Capota 2020	HCM; *N* = 91	N/A	N/A	- No results on anxiety and depression	N/A
McGorrian 2013	AR ICC; *N* = 316	HADS	PW	- Psychological wellbeing is more significant between families than within families, meaning that some families may be inherently predisposed to greater levels of anxiety and depression - ↑ age → ↑HADSd - 19,2% clinical anxiety signals distress	- Lower educational level - Older age - Single/separated - Closely related to family proband
Ingles 2013	ICC; *N* = 409	N/A	N/A	- No results on anxiety and depression	N/A
Hickey 2014	LQTS, BrS, HCM, DCM; *N* = 58	HADS;IPQ	PW	- Similar to other studies. No LT neg conseq of undergoing genetic testing - No correlation found between pos test and PW - Having a positive cardiac genetic diagnosis does not negatively affect overall wellbeing or illness perceptions except for bodily pain domain of the SF-36	- Only on bodily pain - ICD shock→altered illn perc and ↑perc risk - ICD shock→ ↑ IPQ (conseq and emo repr); ↑perc risk, ↑anxiety, ↑depr
Ingles 2015	HCM; *N* = 486	HADS	PW	- ↓understanding of HCM - ↓satisfaction MCS; ↑symptoms; - Conclusion: predictive genetic test for adult onset disease does not lead to long term impairment of PD	- ↑comorbidities; ; ↓income→ ↑ depression; ↓specialized clinic; ↑HADSa→ ↓ medical adherence - Age, MCS non white - Discussion: health outcomes, ↑edu ↑income; attending spec clinic; ↓SES; ↓PW
Brouwers 2015	NCCM(45), FH(43), acq DCM (42); *N* = 130	GAD-7/PHQ-9	Anxiety, depression	- NCCM vs patients FH→ ↓ MCS,↑ anxiety/depression, and ↑PD - NCCM vs DCM acquired→ PD scores are the same	- ↑comorbidity ↑ICD implant ↓age, ↓education
Brothers 2021	AR ARVC; *N* = 73	IES-15	PD	- No significant differences in the scores between baseline, 6 M, 1Y	N/A
Richardson 2018	CPVT; *N* = 54	HADS; IES-R;PAGIS	PW	- Clinical diagnosis +genetic diagnosis ○ 1)Impaired Ψ adaptation ○ 2)↑anxiety - Genetic diagnosis : low PAGIS, ↑anxiety	N/A

This table lists all findings/results found in the included studies from psychological surveys.

*SF-36 MCS* MOS Short form 36 Mental Component Score, *HADS* Hospital Anxiety and Depression Score, *GAD-7* Generalized Anxiety Disorder Scale, *PHQ-9* Patient Health Questionnaire, *IES-15* IES-R Impact of Event Scale, *CAQ-18* Cardiac Anxiety Questionnaire-18, *IPQ-R* The Illness Perception Questionnaire-revised, *BGCSE* The Bergen Genetic Counselling Self-Efficacy scale, *PAGIS* Psychological adaptation to genetic information scale.

Physical and mental health differed according to disease status (i.e., dependent on the cardiac condition and its severity). Symptomatic HCM patients with an underlying genetic cause reported poorer physical health and more debilitating symptoms than patients with a clinical congenital long QT syndrome with an underlying genetic cause, at-risk relatives, and the general population [2, 34, 38]. A cross-sectional study of patients with HCM with an underlying genetic cause reported the lowest scores on physical health in patients who experienced both atypical and exertional pain, and reported significantly lower scores for patients with syncope [38]. Disease duration (i.e., longer time); higher avoidance levels (i.e., cardio-protective avoidance); and being poorly adjusted to ICC were associated with limited physical functioning [19] and poor health [34]. Raised levels of general anxiety are strongly related to avoidance and fear, two factors of heart-focused anxiety [3]. When compared to scores of individuals at-risk or the general population, studies reported higher levels of clinical anxiety [16–52%] and depression scores [8.3–28%] in patients with a clinical diagnosis of ICC [18, 39].

Table 4 lists determinants associated with lower HRQoL. Experiencing clinical symptoms (i.e., chest pain) was significantly associated with higher levels of depression [3, 18, 38, 43]. Lower education [32, 40], single civil status [33], having children [3], and being a woman [33, 40] were sociodemographic risk variables associated with poorer HRQoL.

**Table 4:** Determinants associated with HRQoL.

This table displays all determinants associated with lower HRQoL as well as protective factors identified in the different studies.

*SCD* Sudden cardiac death, *SES* socio-economic status.

Predisposing factors like uncertainty about the genetic screening status of relatives (i.e., no disclosure in the family) and having a close relationship with the person who died of sudden cardiac death [3, 19] were associated with higher levels of general distress, anxiety, and depression.

Older age, clinical diagnosis [2, 34, 41], female biological sex [2, 9, 18, 19, 32–34, 36, 38, 40, 41, 43], comorbidity [9, 32, 41], the presence of symptoms [18, 34, 38, 43], and a higher perceived risk of sudden cardiac death [3, 18, 19, 39] indicated poorer physical health.

Protective factors associated with lower levels of fear and depression include younger age [9, 38] being referred by a specialized clinic, and procedural satisfaction with genetic counseling [9, 44]. Patients with a better understanding of their disease tended to be better adjusted and reported fewer health-related worries [9, 19, 38, 44].

## Discussion

This systematic review provided an overview of PROMs used in patients with ICC and which domains these PROMs assess. While we identified 14 studies using a generic PROM, only 3 used a disease-specific PROM. Even though these tools are fit to measure generic concepts like health status and a disease-specific indication of symptomatology burden, they are not tailored to a specific hereditary condition (i.e., lacking utility and harm arising from genetic testing). Hereditary issues are critical components of ICC and have proven to enhance fears [10, 45, 46] and are suspected to be detrimental to the quality of life.

To be able to use PROMs as a guide for clinical care, the PROM needs to address the needs and preferences of the specific patient group [47]. The management of ICC is complicated and can create a lot of uncertainty, with significant symptom burdens and high levels of psychological distress noted in patients. Poorer mental health places individuals at greater risk for various problems, including impaired HRQoL. Overall, the studies included in this review showed significant impairment in all health status domains and higher levels of general anxiety and depression in patients with a clinical diagnosis of ICC. Prevalence rates in this systematic review of clinically significant anxiety levels ranged from 17% to 47%, and depression rates ranged from 8% to 28%, which is three to five times higher compared with general population controls—these elevated figures of psychological morbidity warrant clinical attention from cardiologists, clinical geneticist and genetic counselors.

Of note, there were several methodological observations, overall studies addressed opportunistic methodology descriptions, and few studies described protocols. All studies were conducted in high-income countries, and demographic data showed a generally low diverse participant population. Participants were predominantly male, white, highly educated, and married. This bias in the samples can influence the reported results. The risk of bias was high or unclear in 75% of the studies meaning that some doubt or even an apparent bias exists, which weakens confidence in the stated study results. Further, we noted that most studies were cross-sectional; more prospective studies are needed to investigate the long-term consequences of disease status on HRQoL in ICC. Many studies used self-constructed scales (i.e., PES HCM, BGCSE) to identify subgroups at risk for psychological distress or impairment in HRQoL at different times. These tools provide a general indication of distress symptomatology over a specific period but need to be validated, which makes it hard to compare results across studies. Standardized and well-validated measures are needed to address the hereditary aspects of ICC.

It is challenging to decide which measure fits best to assess HRQoL in this heterogeneous ICC population (Table S2). Findings of studies reporting the prevalence of depression, anxiety, general well-being, and HRQoL vary because of different assessment methods, which often were not explained enough or used unvalidated instruments. The wide range of study outcome domains (i.e., HRQoL, psychological morbidity, health status, physical health, heart-focused anxiety, and illness perceptions) demonstrates the discordance over what an ICC-PRO should measure. The present review revealed there is currently no standardized, comprehensive, universally accepted PROM instrument for cardiogenetics that addresses the complexity of heredity issues in addition to physical, social, and emotional HRQoL.

A disease-specific PROM can be essential in implementing patient-centered care in cardiogenetics. Several domains of cardiogenetic counseling have the potential for PROM assessment. Besides diagnosis and risk assessment, genetic and cardiac counseling emphasizes goals to educate patients, support them to adapt to genetic and disease-related information and empower patients to make informed decisions in their healthcare path [46]. There is now increasing recognition that PROMs have the potential as instruments that guide clinical care, support clinical decision-making, monitoring treatment effects and disease progression. In patient-centered care, this latter role of PROMs is envisioned. A PROM must be responsive to ICC patients’ preferences, needs, and values to guide clinical care [47]. PROMs nowadays measure patients’ health status rather than the patient”s experience. Future studies should investigate how to integrate these hereditary-related items in a PROM.

## Conclusion

Patients with ICC note elevated levels of psychological morbidity compared to the general population, due to both heart disease-related problems and heredity-specific concerns. This review indicates that up until now, PROMs used in ICC address “overall HRQoL” and are predominantly generic. We propose to develop a disease-specific PROM for the cardiogenetic clinic to evaluate heritability and disease-related factors in ICC patients and to optimize patient-centered care.

### Supplementary information

supplemental material PROMs in cardiogenetics

## Data Availability

The data underlying this article are available in the article and its online supplementary material.
