# Supplementary material for: Are disease-specific patient-reported outcomes measures (PROMs) used in cardiogenetics? A systematic review
Source: Eur J Hum Genet. 2023 Dec 14;32(6):607–18. doi: 10.1038/s41431-023-01510-w (PMC11153546; doi:10.1038/s41431-023-01510-w)
Supplement: Supplementary file 1 — supplemental material PROMs in cardiogenetics [file 41431_2023_1510_MOESM1_ESM.docx]

# Are Disease-Specific Patient-reported Outcomes Measures (PROMs) used in Cardiogenetics? A Systematic Review.

Saar van Pottelberghe MSc^1,2*^, Nina Kupper PhD^3^, Esther Scheirlynck MD^2,4^, PhD, Ahmad S. Amin MD, PhD^2,5^, Arthur A.M. Wilde MD, PhD^2,5^, Nynke Hofman PhD^2,5^ Edward Callus PhD^2,6,7^, Ruth Biller MD^2a,8^, Julie Nekkebroeck PhD^1,2^, Sonia Van Dooren PhD^1,2,9^, Frederik Hes MD, PhD^1,2^ & Saskia N. van der Crabben MD, PhD^2,10^.

**Affiliations**

^1^ Clinical Sciences, research group Reproduction and Genetics, Centre for Medical Genetics, Universitair Ziekenhuis Brussel (UZ Brussel), Vrije Universiteit Brussel (VUB), Laarbeeklaan 101, 1090 Brussels, Belgium.

^2^ Member of the European Reference Network for rare, low prevalence, and/or complex diseases of the heart: ERN GUARD-Heart.

^2a^ European Patient Advocacy Group of the European Reference Network for rare, low prevalence, and/or complex diseases of the heart: ERN GUARD-Heart.

^3^ Center of Research on Psychological disorders and Somatic diseases; Department of Medical & Clinical Psychology, Tilburg University, Tilburg, the Netherlands.

^4^ Cardiology department, Universitair Ziekenhuis Brussel – Vrije Universiteit Brussel, Brussels, Belgium.

^5^ Amsterdam UMC location University of Amsterdam, Department of Cardiology, Meibergdreef 9, Amsterdam, the Netherlands; Amsterdam Cardiovascular Sciences, Heart Failure and arrhythmias, Amsterdam, the Netherlands^6^ Clinical Psychology Service, IRCCS Policlinico San Donato Research and University Hospital, San Donato Milanese, Milan, Italy.

^7^ Department of Biomedical Sciences for Health, University of Milan, Milan, Italy.

^8^ ARVC-Selbsthilfe e.V., ARVC Patient Association, Munich, Germany.

^9^ Clinical Sciences, research group Reproduction and Genetics, Brussel Interuniversity Genomics High Throughput core (BRIGHTcore), Universitair Ziekenhuis Brussel (UZ Brussel), Vrije Universiteit Brussel (VUB), Laarbeeklaan 101, 1090 Brussels, Belgium.

^10^ Department of Human Genetics, Amsterdam UMC, University of Amsterdam, Amsterdam, The Netherlands.

*Corresponding author

Saar Van Pottelberghe, Center of Medical Genetics, University Hospital Brussels, Laarbeeklaan 101, 1090 Brussels, Belgium.

Email: [saartje.vanpottelberghe@uzbrussel.be](mailto:saartje.vanpottelberghe@uzbrussel.be)

Telephone number: 0032 470180230 / ORCID 0000-0001-9531-9601

Supplemental material

**Method:**

**S1** Risk of Bias quality assessment tool with instructions

**Results:**

**Table S1** Summary of rated biases for all the studies

Note: This figure details per risk of bias domain the score per included study. High risk= red; unclear risk=yellow; low risk= green

**Table S2** PROMS, psychological and physical measures used in the included studies.

Note: This table lists all measures used in the included studies. All studies used a PROM, and sometimes an additional PREM. Further studies used different psychological surveys to assess wellbeing, anxiety and depression levels, as well as the physical component score of either SF-36/12 and KCCQ were used to interpret health status, general perceived health and HRQoL

**Supplement 1 ROB assessment tool [31]**

**RECORD NUMBER: REVIEWER: DATE OF REVIEW:**

| CRITERIA^a^ | YES | | NO | | | UNCLEAR | | N/A |
| --- | --- | --- | --- | --- | --- | --- | --- | --- |
| 1. Are critical inclusion/exclusion criteria clearly stated (does not require the reader to infer)? |  | |  | | |  | |  |
| 1. Did the study apply inclusion/exclusion criteria uniformly to all comparison groups? |  | |  | | |  | |  |
| 1. Does the analysis control for baseline differences between groups? |  | |  | | |  | |  |
| 1. Does the design or analysis control account for important confounding and modifying variables through matching, stratification, multivariable analysis, or other approaches? |  | |  | | |  | |  |
| 1. Was the response rate reported by the researchers? |  | |  | | |  | |  |
| - **SELECTION BIAS (YES)^b^** | ** low risk** | | ** high risk** | | | ** unclear risk** | | |
| 1. Is the length of follow-up the same for all groups? |  | |  | | |  | |  |
| 1. Did attrition from any group remain below 20% for FU<1 year or 30% for FU>1 year? |  | |  | | |  | |  |
| 1. Did attrition differences between groups remain below 20%? |  | |  | | |  | |  |
| 1. If attrition (overall or differential non-response, drop-out, loss to follow-up, or exclusion of participants) was a concern, were missing data handled appropriately (e.g., intention-to-treat analysis, sensitivity analysis, or imputation)? |  | |  | | |  | |  |
| - **ATTRITION BIAS (YES)^b^** | ** low risk** | | ** high risk** | | | ** unclear risk** | | |
| 1. Were psychological determinants assessed/defined using valid and reliable measures, implemented consistently across all study participants? |  | |  | | |  | |  |
| 1. Was patient centred care assessed or defined using valid and reliable measures, implemented consistently across all study participants? |  | |  | | |  | |  |
| 1. Were confounding variables assessed using valid and reliable measures, implemented consistently across all study participants? |  | |  | | |  | |  |
| - **INFORMATION BIAS (YES)^b^** | ** low risk** | | ** high risk** | | | ** unclear risk** | | |
| 1. Were the potential outcomes pre-specified by the researchers? |  | |  | | |  | |  |
| Are all pre-specified outcomes reported? |  | |  | | |  | |  |
| - **REPORTING BIAS (YES)^b^** | ** low risk** | | ** high risk** | | | ** unclear risk** | | |
| 1. Was the sample size sufficiently large to detect a clinically significant difference of 5% or more between groups in at least one primary outcome measure? |  | |  | | |  | |  |
| 1. Are the statistical methods used to assess the primary benefit outcomes appropriate to the data? |  | |  | | |  | |  |
| - **LACK OF PRECISION (YES)^b^** | ** low risk** | | ** high risk** | | | ** unclear risk** | | |
| SUMMARY OUTCOME | |  | | |  | |  | |
| - RISK OF BIAS^c^ | | ** low risk** | | | ** high risk** | | ** unclear risk** | |
| **ADDITIONAL CRITERION^a^** | | **YES** | | **NO** | | **UNCLEAR** | | **N/A** |
| 1. Was patient centred care measured objectively (e.g., MEMS for measuring medication adherence)? | |  | |  | |  | |  |
| - NO OBJECTIVE PATIENT CENTRED CARE MEASURE^b^ | |  low risk | |  high risk | |  unclear risk | | |

^a^ Each criterion is evaluated for a judgment of ‘yes’, ‘no’ or ‘unclear’.

^b^ Each type of bias is judged based on the criteria:

- - Low risk of bias if all criteria are judged with ‘yes’
  - High risk of bias if one or more of the criteria are judged with ‘no’
  - Unclear risk of bias if one or more of the criteria are judged with ‘unclear’ but none with ‘yes’

^c^ Assess a summary outcome (across bias domains) within studies:

| RISK OF BIAS | INTERPRETATION | WITHIN A STUDY | ACROSS STUDIES |
| --- | --- | --- | --- |
| Low risk of bias | Plausible bias unlikely to seriously alter the results | Low risk of bias for all key domains of bias | Most information is from studies at low risk of bias |
| Unclear risk of bias | Plausible bias that raises some doubt about the results | Unclear risk for one or more key domains of bias | Most information is from studies of low or unclear risk of bias |
| High risk of bias | Plausible bias that seriously weakens confidence in the results | High risk of bias for one or more key domains of bias | The proportion of information from studies at high risk of bias is sufficient to affect the interpretation of the results |

| **Table S1 Summary of rated biases for all the studies** | | | | | | | |
| --- | --- | --- | --- | --- | --- | --- | --- |
| **Study-ID** | **Total numbers of participants** | **Selection** | **Attrition** | **Information** | **Reporting** | **Lack of Precision** | **RISK of Bias** |
| Cox 1997 | 137 | HIGH RISK | N/A | LOW RISK | LOW RISK | LOW RISK | HIGH RISK |
| Steptoe 2000 | 60 | HIGH RISK | N/A | LOW RISK | LOW RISK | UNCLEAR RISK | HIGH RISK |
| Hamang 2012 | 173 | LOW RISK | HIGH RISK | UNCLEAR RISK | LOW RISK | LOW RISK | HIGH RISK |
| Ingles 2012 | 54 | HIGH RISK | HIGH RISK | UNCLEAR RISK | UNCLEAR RISK | UNCLEAR RISK | HIGH RISK |
| Ingles 2015 | 486 | HIGH RISK | N/A | UNCLEAR RISK | UNCLEAR RISK | LOW RISK | HIGH RISK |
| Brothers 2021 | 73 | HIGH RISK | UNCLEAR RISK | LOW RISK | UNCLEAR RISK | HIGH RISK | HIGH RISK |
| Pedrosa 2010 | 84 | UNCLEAR RISK | N/A | UNCLEAR RISK | LOW RISK | LOW RISK | UNCLEAR RISK |
| Huff 2012 | 24 | UNCLEAR RISK | N/A | UNCLEAR RISK | LOW RISK | LOW RISK | UNCLEAR RISK |
| Ingles 2013 | 409 | UNCLEAR RISK | N/A | LOW RISK | LOW RISK | LOW RISK | UNCLEAR RISK |
| Brouwers 2015 | 130 | UNCLEAR RISK | N/A | UNCLEAR RISK | LOW RISK | UNCLEAR RISK | UNCLEAR RISK |
| Richardson 2018 | 54 | UNCLEAR RISK | N/A | LOW RISK | LOW RISK | LOW RISK | UNCLEAR RISK |
| Hickey 2014 | 58 | LOW RISK | N/A | LOW RISK | UNCLEAR RISK | UNCLEAR RISK | UNCLEAR RISK |
| Christiaans 2009 | 228 | LOW RISK | N/A | LOW RISK | LOW RISK | LOW RISK | LOW RISK |
| Hamang 2010 | 127 | LOW RISK | N/A | LOW RISK | LOW RISK | LOW RISK | LOW RISK |
| Hamang 2011 | 126 | LOW RISK | N/A | LOW RISK | LOW RISK | LOW RISK | LOW RISK |
| McGorrian 2013 | 316 | LOW RISK | N/A | LOW RISK | LOW RISK | LOW RISK | LOW RISK |
| Capota 2020 | 91 | LOW RISK | N/A | LOW RISK | LOW RISK | LOW RISK | LOW RISK |

| **Table S2 PROMS, psychological; and physical measures** | | | | | | | | | |
| --- | --- | --- | --- | --- | --- | --- | --- | --- | --- |
| **Study ID** | **Disease/ sample size** | **PROM** | | **+ PREM** | | **+ Psychological surveys** | | | **+ Physical health** |
|  |  | **Generic** | **Specific** | **Specific** | **Satisfaction** | **Anxiety/depression** | **Impact** | **Adaptation** | **Limitations** |
| Cox 1997 | HCM; N=137 | SF-36 | - | PES | PSS | HADS | - | - | SF-36 PCS |
| Steptoe 2000 | DCM; N=60 | SF-36 | - | PES |  | HADS |  | - |  |
| Christiaans 2009 | HCM; N=228 | SF-36 | - | PES | - | - | IPQ | - | SF-36 PCS |
| Hamang 2010 | LQTS, HCM + presymptomatic LQTS, HCM; N=127 | SF-36 | - | - | - | - | - | - | SF-36 PCS |
| Pedrosa 2010 | HCM (n=84); asymptomatic controls (n=42); N=126 | - | MLHFQ | - | - | - | - | - |  |
| Hamang 2011 | LQTS, HCM + presymptomatic LQTS, HCM; N=126 | SF-36 | - | - | - | HADS | CAQ-18 | - | SF-36 PCS |
| Hamang 2012 | LQTS, HCM + presymptomatic LQTS, HCM; N=173 | SF-36 | - | - | - | - | CAQ-18 | BGCSE | SF-36 PCS |
| Ingles 2012 | ICC (33)+ presymptomatic ICC(21); N=54 | SF-36 | - | - | - | - | - | - | SF-36 PCS |
| Huff 2013 | HCM; N=24 | - | KCCQ | - | - | - | - | - | KCCQ PCS |
| McGorrian 2013 | presymptomatic ICC; N=316 | SF-12 | - | - | - | HADS | - | - | SF-12 PCS |
| Ingles 2013 | ICC; presymptomatic ICC; N=409 | SF-36 | - | - | - | - | - | - | SF-36 PCS |
| Hickey 2014 | LQTS, BrS, HCM, DCM; N=58 | SF-36 | - | PES | - | HADS | IPQ | - | SF-36 PCS |
| Ingles 2015 | HCM + presymptomatic HCM; N=486 | SF-36 | - | PES | SWD | HADS |  | - |  |
| Brouwers 2015 | NCCM(45), FH(43), acq DCM (42); N=130 | SF-12 | - | - | - | GAD-7, PHQ-9 | - | - | SF-12 PCS |
| Richardson 2018 | CPVT; N=54 | SF-36 | - | PES | - | HADS | IES-R | PAGIS | SF-36 PCS |
| Capota 2020 | HCM; N=91 | - | KCCQ | - | - | - | - | - | KCCQ PCS |
| Brothers 2021 | presymptomatic ARVC; N=73 | SF-36 | - | - | - | - | IES-15 |  | SF-36 PCS |
| **Total** | **N=2648** | **n= 14 (82%)** | **n=3 (18%)** | **n=6 (35%)** | **n=2 (12%)** | **n=8 (50%)** | **n=6 (37%)** | **n=2 (13%)** | **n=14 (82%)** |
